# Revisiting Oxidative Stress and the Use of Organic Selenium in Dairy Cow Nutrition

**DOI:** 10.3390/ani9070462

**Published:** 2019-07-19

**Authors:** Peter F. Surai, Ivan I. Kochish, Vladimir I. Fisinin, Darren T. Juniper

**Affiliations:** 1 Department of Microbiology and Biochemistry, Faculty of Veterinary Medicine, Trakia University, 6000 Stara Zagora, Bulgaria; 2 Moscow State Academy of Veterinary Medicine and Biotechnology Named after K.I. Skryabin, 109472 Moscow, Russia; 3 Department of Animal Nutrition, Faculty of Agricultural and Environmental Sciences, Szent Istvan University, H-2103 Godollo, Hungary; 4 All-Russian Institute of Poultry Husbandry, 141311 Sergiev Posad, Russia; 5 Animal, Dairy, Food Chain Sciences, School of Agriculture, Policy and Development, University of Reading, Earley Gate, Reading RG6 6AR, UK

**Keywords:** oxidative stress, organic selenium, nutrition, dairy, antioxidant

## Abstract

**Simple Summary:**

Among feed-derived antioxidants, selenium (Se) was shown to have a special place as an essential part of 25 selenoproteins identified in animals. Organic Se, in the form of selenomethionine (SeMet), has been reported to be a much more effective Se source when compared with mineral forms such as sodium selenite or selenate. There is a growing body of evidence that demonstrates that organic Se has a number of benefits, particularly in dairy and beef animals; these include improved selenium and antioxidant status, and better Se transfer via the placenta, colostrum, and milk to the newborn. However, there is a paucity in the data concerning molecular mechanisms of SeMet assimilation, metabolism and selenoprotein synthesis regulation in ruminant animals, and as such, further investigation is required.

**Abstract:**

In commercial animals production, productive stress can negatively impact health status and subsequent productive and reproductive performance. A great body of evidence has demonstrated that as a consequence of productive stress, an overproduction of free radicals, disturbance of redox balance/signaling, and oxidative stress were observed. There is a range of antioxidants that can be supplied with animal feed to help build and maintain the antioxidant defense system of the body responsible for prevention of the damaging effects of free radicals and the toxic products of their metabolism. Among feed-derived antioxidants, selenium (Se) was shown to have a special place as an essential part of 25 selenoproteins identified in animals. There is a comprehensive body of research in monogastric species that clearly shows that Se bioavailability within the diet is very much dependent on the form of the element used. Organic Se, in the form of selenomethionine (SeMet), has been reported to be a much more effective Se source when compared with mineral forms such as sodium selenite or selenate. It has been proposed that one of the main advantages of organic Se in pig and poultry nutrition is the non-specific incorporation of SeMet into general body proteins, thus forming an endogenous Se reserve that can be utilized during periods of stress for additional synthesis of selenoproteins. Responses in ruminant species to supplementary Se tend to be much more variable than those reported in monogastric species, and much of this variability may be a consequence of the different fates of Se forms in the rumen following ingestion. It is likely that the reducing conditions found in the rumen are responsible for the markedly lower assimilation of inorganic forms of Se, thus predisposing selenite-fed animals to potential Se inadequacy that may in turn compromise animal health and production. A growing body of evidence demonstrates that organic Se has a number of benefits, particularly in dairy and beef animals; these include improved Se and antioxidant status and better Se transfer via the placenta, colostrum, and milk to the newborn. However, there is a paucity in the data concerning molecular mechanisms of SeMet assimilation, metabolism and selenoprotein synthesis regulation in ruminant animals, and as such, further investigation is required.

## 1. Introduction

In commercial dairy and beef production, a range of stresses are responsible for economic losses associated with the decreased productive and reproductive performance of cows. It has been shown that at the molecular level, nutritional, technological, environmental and internal stresses lead to the overproduction of free radicals, the disturbance of the redox balance, and oxidative stress [1,2]. It is well known that oxidative stress (an imbalance between free radical production and their detoxification) has a number of detrimental consequences affecting immune and reproductive systems, as well as major parameters of animal growth, development and general health [3,4]. Therefore, the antioxidant defense network developed during animal evolution is responsible for the maintenance of redox balance in cells and tissues, preventing the detrimental consequences of commercially relevant stresses. In the body, all antioxidants work collectively together, and each has its own specific function. Within this collective antioxidant system, selenium (Se) is considered to be of paramount importance [5,6]. Indeed, there are currently 25 selenoproteins identified in animal tissues, and more than half are directly or indirectly involved in the maintenance of the body redox balance and antioxidant defense [6]. However, there is a need for a deeper understanding of the molecular mechanisms of Se uptake into the body/cells and its subsequent usage for maintaining animal health. It has been fairly well established in a number of animal species that Se bioavailability depends on the form of dietary Se offered [7,8,9]. In ruminant species the assimilation of Se from the diet can be dependent upon rumen conditions that can substantially decrease the availability of mineral forms of Se, in particular sodium selenite [10]. Organic forms of Se do not appear to suffer the same ruminal fate as mineral forms, and as such, would be more available for utilization by dairy cows [11,12]. Therefore, the aim of the current review is to critically analyze existing knowledge on the role of organic Se in dairy cow nutrition with specific emphasis on commercially relevant stresses.

## 2. Free Radicals and Reactive Oxygen and Nitrogen Species

Free radicals are atoms or molecules with one or more unpaired electrons that are characterized by high reactivity. They are capable of damaging all types of biologically relevant molecules including DNA, proteins, lipids and carbohydrates [13]. Collective terms such as reactive oxygen species (ROS) and reactive nitrogen species (RNS) are commonly accepted [8] and include not only the oxygen or nitrogen radicals, but also some non-radical reactive derivatives of oxygen and nitrogen (Table 1).

It has been shown that ROS and RNS are produced as by-products of the body’s normal metabolic activity, and as part of the immune system’s protective activity against invading microorganisms. Detrimental consequences of ROS on cellular metabolism are due to their participation in lipid peroxidation and protein oxidation reactions [15], which can result in substantial damage to cells. Polyunsaturated fatty acids (PUFA) are integral to cell membranes and are responsible for fluidity and permeability of this membrane, as well as being involved in a number of vital biological processes, including signaling, growth, development and survival. However, PUFAs are susceptible to oxidation, the degree of oxidation being proportional to the number of double bounds in the molecule. It has been shown that lipid peroxidation is closely associated with various disease states and decreased productive and reproductive performance in farm animals, including dairy cows [16,17].

Protein and DNA can also be damaged by ROS. The complex structure of proteins and the presence of a variety of oxidizable functional groups in amino acids (e.g., the sulfur group in cysteine and methionine) explain their high susceptibility to oxidative damage. Consequently, alteration of the tertiary structure of a protein due to direct oxidation of a specific amino acid, or as a result of cleavage of the protein backbone, may result in modification of the biological activity of the altered protein. The degree of protein damage has been shown to depend on many different factors [18] and direct oxidation of cysteine and methionine residues in proteins are considered to be major consequences of oxidative stress-induced changes to protein activity and function [19].

Oxidation, methylation, deamination and depurination are the four most important endogenous processes causing significant DNA alteration. The chemistry of the interactions between ROS and DNA is very complex, and negative effects of ROS action are associated with damage to bases, sugar lesions, single strand-breaks, basic lesions and DNA-nucleoprotein cross-links [20]. DNA damage leads to a number of detrimental effects that include carcinogenesis, aging, neurodegenerative disease, consequences of mutations, genome instability and altered cell signaling. In addition, lipid peroxidation-derived aldehydes and their exocyclic DNA adducts can result in various mutations [21]. For example, the spontaneous mutation rate in humans is estimated to be quite high, comprising about 5 × 10^−11^ mutations per base per cell division [22]. However, consequences of DNA oxidation/damage for animal health maintenance, including its effects on immunocompetence, and the productive and reproductive abilities of cows and other farm animals, have not been well established and require further research. Oxidative stress in farm animals, including dairy cows, has been shown to compromise their health status and lead to immunosuppression, decreased productive and reproductive performance [23,24,25]. However, depending on type and concentration, ROS can be beneficial or detrimental to cellular physiology. Recently ROS have been suggested to be essential drivers of evolution and survival during Earth’s history [26]. Furthermore, ROS formation is thought to be an important evolutionarily conserved process playing a regulatory role in cell signaling mechanisms, as well as in adaptation of cellular defense networks to various stresses, including microbial invasion [27].

## 3. Biological Antioxidant Systems

**Figure 1:**
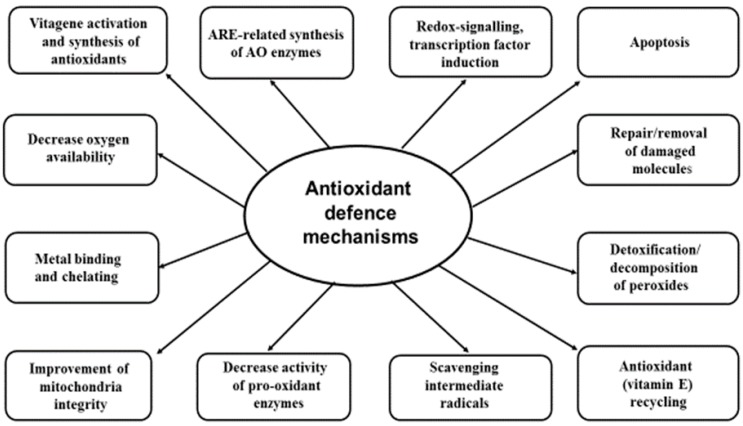
Antioxidant defense mechanisms in the body (Adapted from [6,30]).

Antioxidant systems are believed to have evolved as a means of surviving in an oxygenated atmosphere by dealing with free radicals and the toxic products of their metabolism. Animal antioxidant defense mechanisms are based on the synthesis of numerous biological antioxidants that include the antioxidant enzymes, glutathione, thioredoxin, and coenzyme Q [6,28]. There is also a range of dietary antioxidants which can be provided in feed, which include vitamin E, carotenoids, polyphenolics, and Se (as a precursor to selenoproteins). Under stress conditions, the internal antioxidant system network alone cannot deal properly with excess ROS formation and requires additional help from dietary antioxidant sources provided via feed/water. Vitamin E and Se are major feed-derived antioxidants [6,29]. Antioxidant defense mechanisms in the body are summarized in Figure 1.

Antioxidant defence strategy is based on several lines of defence. These include various antioxidants that directly scavenge ROS and RNS, detoxifying products of their metabolism and repairing damaged molecules. Additional mechanisms include vitamin E recycling, binding Fe^2+^ and Cu^+^, and decreasing oxygen availability. Mechanisms that prevent ROS/RNS formation (mitochondria integrity maintenance, decreased activity of pro-oxidant forming enzymes) are important elements of the antioxidant defence strategy. Redox signaling and transcription factor induction with ARE-related synthesis of protective molecules substantially contribute to antioxidant defence mechanisms. Vitagene activation and improvement of adaptability to stress as well as apoptosis of terminally damaged cells are considered a recent addition to the integrative antioxidant defence system of the body.

It is important to mention that ROS and RNS are no longer viewed as just toxic by-products of cellular metabolism and their role in regulating cell signaling pathways is recognized. It is believed that oxidation-reduction (redox)-based regulation of gene expression is a fundamental regulatory mechanism of cell signaling [31]. Indeed, the field of redox signaling and signal transduction is a very rapidly developing area of molecular biology. It is well accepted that redox signaling is a key element in maintaining physiological homeostasis and disbalance in redox homeostasis leads to compromised immunity and subsequent disease development [32], thus decreasing productive and reproductive performance. Redox-signaling pathways use ROS as signaling molecules to activate vitagenes, which are responsible for adaptation to stress [30], as well as genes responsible for regulation of growth, differentiation, proliferation and apoptosis [6,33]. The term “vitagene” was introduced in 1998 by Rattan [34] to describe several genes that are strictly involved in preserving cellular homeostasis during stress conditions and the vitagene family includes heat shock proteins, the thioredoxin system, the glutathione system and sirtuins [35]. The products of the above-mentioned genes actively operate in detecting and controlling diverse forms of stress and cell injuries and vitagene activation, with the following synthesis of a range of protective antioxidant molecules, is the central event in a stress adaptation. The vitagene concept found its acceptance in medical sciences [36,37,38] and poultry sciences [39], however, the protective role of vitagenes in cow adaptation to stress requires further investigation.

Furthermore, antioxidant defense systems and cellular redox balance are shown to be controlled by a battery of transcriptional factors that include nuclear-related factor-E2 (Nrf2), nuclear factor-kappa B (NF-κB), peroxisome proliferator-activated receptors (PPARs), peroxisome proliferator-activated receptor gamma coactivator 1-alpha (PGC-1α), forkhead box O (FoxO), mitogen activated protein kinase (MAPK), and activator protein 1 (AP1). They regulate redox status by modulating ROS-generating enzymes and additional synthesis of antioxidant enzymes (Nrf2) and promoting inflammation (NF-κB) in a cooperative and interactive way, being critically important for the animal’s adaptation to environmental, technological and nutritional stress [40,41,42,43].

## 4. Oxidative Stress in Dairy Cattle

Significant advances have been made over the last 30 years with respect to efficiencies of production in dairy cattle. However, there are several problems in dairy production that require a solution; these include immunity (poor udder health [44], poor reproduction (low conception rate, retained placenta, metritis, cystic ovaries [45,46,47] and reduced calf viability in early postnatal life [48].

In the commercial conditions of milk production, there are four main groups of stressors: physical, chemical, biological and psychological [49]. Indeed, fluctuations in ambient temperature, mechanical injuries, irregularities in habits and or routines, nutrient disbalance, mycotoxins contamination of feed, microbial and viral infections, etc., are important stress-factors contributing to oxidative stress which can potentially decrease productive and reproductive performance and compromise animal health. In general, there is a range of various biomarkers used for detection of OS and their advantages and limitations are summarised in Table 2.

It is necessary to emphasize that there is no single test that would give an ultimate answer to what the optimal oxidative status of an animal should be, and therefore a combination of various tests is preferable. For example, the ratio of pro-oxidant to antioxidant capacity, known as the oxidative status index (OSi), was suggested as a tool to assess the redox status and possible oxidative stress in dairy cattle [59]. However, it is important to measure macromolecule damage (e.g., lipid and/or protein oxidation products) that can occur due to free radical overproduction to make a conclusion about oxidative stress in farm animals [24].

The periparturient period (the last 1 to 2 months of gestation and the first few months post-partum), and particularly the transition period (3 weeks before to 3 weeks after parturition) of dairy cattle are associated with dramatic changes in metabolism and immune defense mechanisms that predispose animals to an increased risk of disease [60]. It is generally accepted that transition cows are susceptible to oxidative stress [54], which can be exacerbated by several stress factors, including environmental stress (e.g., heat stress), nutrition, body condition score, disease challenge, obesity, and increased plasma NEFA concentrations [49,61], as shown in Table 3.

However, stress response is quite a complex issue and depends on many different factors, including stress signaling and adaptation. For example, feeding different amounts of concentrates to dairy cows did not affect major OS markers such as dROM, FRAP, or OSi [75]. According to Celi [76] and Sordillo Mavangira [77] oxidative stress has been implicated in the development of numerous disease states, including, mastitis, acidosis, ketosis, enteritis, pneumonia and respiratory diseases. In dairy cows, oxidative stress is also shown to be associated with the retention of fetal membranes post-calving, as well as disrupting activity of the corpus luteum [78]. It is well appreciated that ROS play a regulatory role in female reproduction, including folliculogenesis, corpus luteum oocyte maturation and feto-placental development via various signaling transduction pathways [79]. However, excessive RONS production and oxidative stress are shown to be involved in the development of various reproductive disorders and in the pathophysiology of complicated pregnancies. In fact, ROS was shown to affect various physiologic functions of the ovary, including ovarian steroid genesis, oocyte maturation, ovulation, formation of blastocysts, implantation, luteolysis and luteal maintenance in pregnancy [80]. Therefore, OS affects the female reproductive system of farm animals at several levels, from oocyte maturation to fertilisation and embryo development [81]. Oocytes and other follicular cells were shown to be sensitive to oxidative damage, leading to the depletion of the ovarian pool of primordial follicles and damaging surviving oocytes [82]. There is a growing body of evidence that OS is implicated in, and may possibly be a cause of, increased embryonic mortality in dairy cows [51]. Furthermore, metabolic stress includes three components: altered nutrient metabolism, oxidative stress, and inflammation [83]. Recent findings related to OS in dairy cows (summarised in Table 3) confirmed the hypothesis that oxidative stress is an important element of modern industrial milk production. It was suggested that lower paraoxonase-1 (PON1) activity in lactating cows in comparison to heifers is a result of increased metabolic efforts during pregnancy and parturition associated with oxidative stress [84]. Recently, it has been shown that prenatal exposure to OS was associated with adverse effects on the offspring that could influence disease susceptibility. In fact, calves born to cows with increased OSi during late gestation showed decreased body weight at birth and throughout the study in comparison to the control animals. Serum OS biomarkers, including ROS and RNS concentrations as well as TNF-α (a proinflammatory cytokine), were shown to be higher in calves exposed to higher maternal OSi when compared with calves born to cows with lower values of these biomarkers [52]. Interestingly, authors also showed compromised immune responses to microbial agonists in calves exposed to higher maternal biomarkers of OS. Signaling through the Nrf2 pathway in cow mammary glands during the postpartum period is considered a key component of adaptive cellular function to maintain proper redox homeostasis [85].

The increased incidence of health problems observed during the periparturient period can be partly a consequence of suboptimal immune responses due to various stress factors/conditions [86]. Indeed, complex interactions between disturbed metabolism and immune function that predispose cows to periparturient diseases deserve more attention [87]. However, molecular mechanisms regulating those changes are still not well defined. It seems likely that increased ROS production and antioxidant defense inadequacy during these critical periods pre and post calving play important roles in immune system compromise and health-related problems [23,47,88].

Indeed, it is well accepted that the transitional period in cows is immunosuppressive and characterized by defective neutrophil and lymphocyte function [89,90]. In addition, the immunosuppression biomarker Toll-Like-Receptor 2 (TLR2) gene was reported to be up-regulated at calving and for the first week after parturition [91]. It was well established that the transition from a non-lactating pregnant status to a non-pregnant lactation status is an important period affecting health, production and profitability of dairy cows. In fact, most infectious diseases and metabolic disorders which include retention of fetal membranes, metritis, and mastitis are associated with this periparturient period [1,92]. It seems likely that the physical and metabolic stresses of pregnancy, calving, and lactation contribute substantially to the decrease in host resistance and any subsequent increase in disease incidence [93]. Indeed, there is an increasing body of evidence that shows that innate and acquired defense mechanisms are lowest between 3 weeks pre-calving to 3 weeks post-calving and usually result in the increased incidence of peripartum diseases such as retained fetal membranes, elevated somatic cell counts, and mastitis [94]. When compared to other species, cow neutrophils are characterized by decreased myeloperoxidase and elastase activities and an absence of α-defensins [95], and probably under stress conditions their function could be further compromised [96], predisposing transition cows to periparturient-related health issues.

## 5. Nutritional Modulation of the Antioxidant Network to Prevent Oxidative Stress

**Figure 2:**
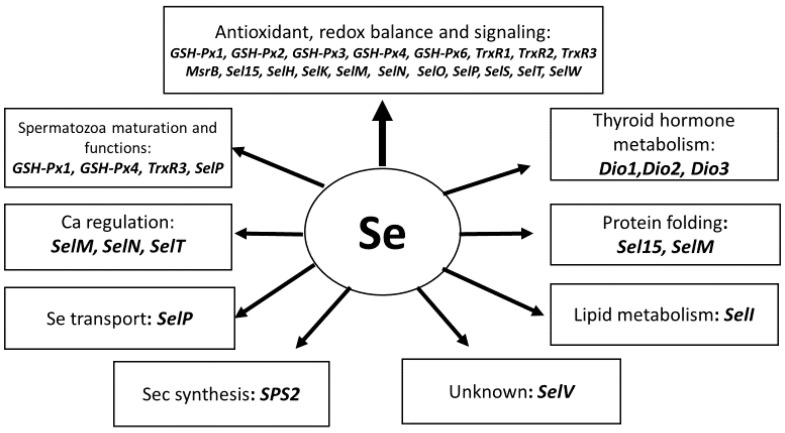
Selenoprotein functions in animals (adapted from [6,103]).

As previously mentioned, there is a range of dietary antioxidants which can be added to the animal diet to improve antioxidant defense. Among them, Se has a special place as a precursor of at least 25 selenoproteins [97], playing important roles in the regulation of vital pathways in the animal body and contributing substantially to the antioxidant defense network [6,94,98]. Selenoproteins are located in various parts of the cell and are involved in a number of physiological important functions, including peroxidase and reductase activities, hormone metabolism, protein folding, redox signaling, selenocysteine synthesis, and Se transport. Importantly, more than half known selenoproteins participate in redox balance maintenance and protection against oxidative stress via peroxidase/reductase activities and redox signaling [99,100,101,102]. Involvement of major selenoproteins in animal metabolism and function are shown in Figure 2 [6,103].

Twenty-five selenoproteins in animals/cows participate in the regulation of a range of various functions in the body. A total of 19 selenoproteins are involved in redox balance maintenance and antioxidant defences. Spermatozoa maturation and function are dependent on the redox balance and GSH-Px4 has a specific function in the spermatozoa structure, being converted from enzymatic to a structural protein. Three Se-dependent deiodinases regulate thyroid metabolism. SelI regulates lipid metabolism and Sel15 and SelM participate in protein folding. SelM, SelN and SelT are involved in Ca metabolism regulation. SelP is an important element of Se transport and SPS2 is responsible for Sec synthesis. The role of SelV in animals is currently unknown.

A common feature of Se nutrition in farm animals is the low concentration of Se in feed ingredients [6,94]. The selenium cycle in the food chain of ruminants starts from soils and includes plant sources ultimately dependent on its assimilation from the soil. Indeed, soils are the major source of Se for plants and therefore for animals eating those plants and humans consuming plant- and animal-derived foods. Selenium concentration in soils varies significantly. The Se content of most soils ranges between 0.1 and 2 mg/kg, with an average concentration of 0.2 mg/kg and great geographical variability [104]. Soil Se exists in various forms, including selenides, elemental Se, selenites, selenates and organic Se compounds [105]. High concentrations of Se are found mainly in sedimentary rocks and shales formed during the cretaceous period, while lower concentrations of Se are characteristic for igneous (volcanic) rock, sandstone, granite and limestone [106]. On the one hand, soils developed under tropic and subtropic conditions (laterite, yellow soil and red soil) contain comparatively high Se levels (>0.3 ppm [107]). On the other hand, soils developed under temperate (warm) steppe and desert conditions (chernozem, chestnut soil, calcic brown soil, desert soil and solonchak) are characterised by moderate Se concentrations (0.14–0.30 ppm). Finally, soils such as brown earth, drab soil, dark brown soil, loessial soils, purple soil, and red drab soil, developed under temperate (warm) humid/sub-humid conditions are quite poor in Se [107]. Furthermore, Se availability to plants depends on many factors including soil pH, the oxidation-reduction potential and mineral composition of the soil, the rate of artificial fertilization and rainfall; therefore, the bioavailability of Se in soils for plants depends more on its form than on its total concentration [108,109]. Indeed, it is generally accepted that environmental conditions and agricultural practices have a major effect on the Se content of various plant feeds.

Since Se levels in soils vary and Se availability to plants depends on many factors [105,110] the general agricultural practice in the world includes Se supplementation of farm animal diets. Therefore, the need for Se supplementation has long been recognized and sodium selenite (SS) or selenate were first approved as Se supplements by the USA FDA in 1974. Nowadays Se has become an important part of vitamin and mineral premixes for farm animals, and although problems of Se deficiency in monogastric animals have been successfully solved, within the commercial dairy industry, there are still reported cases of Se inadequacy/deficiency [94,111]. This issue is further compounded by the fact that Sodium selenite (SS) and selenate are not optimal forms of Se supplementation for ruminant animals, as the fate and utilization of these forms are adversely affected by rumen conditions [10,112,113,114,115].

## 6. Organic Selenium Concept Development

Recent advances in Se biochemistry were associated with an understanding that in major feed ingredients, including grains (wheat, barley, corn, etc.) and oil seeds (soya), Se is found mainly in the organic form SeMet, representing more than 50% of total Se [103,116,117]. It seems likely that during evolution, the digestive system of animals adapted to organic Se supply and as a consequence, it is efficiently assimilated from the diet and used in metabolic processes. Since, chemically, SeMet is very similar to methionine (Met), transport systems in the body do not distinguish between Met and SeMet, and as a consequence SeMet can be taken up from the gut and non-specifically incorporated into tissue proteins, replacing some methionine, and building an endogenous Se reserve within the body [103].

Whilst SeMet is considered a storage form of Se, de novo synthesized selenocysteine (SeCys) is the functional biological form of Se, incorporated at a genetic level into the active centers of selenoproteins. It was suggested that SeMet reserves in the body can be used during stress conditions, when Se requirements increase whilst Se supply decreases due to reduced feed consumption [6,103]. It seems likely that proteosome degradation of body proteins during times of stress may be one mechanism by which accumulated SeMet can be released, acting as an additional source of Se [6,103]. Indeed, ATP- and ubiquitin-independent proteolysis by the 20S proteasome is an important mechanism for the selective degradation of oxidised proteins. Furthermore, the 20S proteasome is characterised by increased proteolytic activity towards oxidised polypeptides, whilst increased GSH-Px1 activity can downregulate basal 20S proteasome activity [118]. The authors suggested that intracellular redox status is responsible for activating or down-regulating the 20S proteasome chymotrypsin-like activity in living cells, thus explaining how Se-reserves in the body in the form of SeMet can be used to enable an animal to better adapt to stress, meeting the requirement for the additional synthesis of selenoproteins to meet increased antioxidant defence requirements that can be associated with stress conditions. Therefore, building Se reserves in the body should be considered an important strategy for dealing with commercially relevant stresses in farm animal production. Since SeMet cannot be synthesized by animals, it has to be supplied within the diet and supplementing commercial animal diets with organic Se supplements is necessary in order to develop this endogenous reserve [6,103].

The immune system and gut health are two of the most affected areas during stress conditions. Recently, stresses in monogastric species were divided into four main categories: environmental, technological, nutritional and internal/biological [6]. Similar stress categories were associated with commercial milk production [61,119] and since it is practically impossible to avoid these stresses in commercial animal production, an important task for animal nutritionists is to balance the diet and provide optimal antioxidant defense.

## 7. Important Features of Selenium Metabolism in Ruminants

The small intestine is the primary site of Se absorption, with no absorption from the rumen [120]. It is necessary to underline that there are several factors affecting the efficiency of Se absorption. These include the form of the element, the amount ingested, and other dietary factors such as calcium, arsenic, cobalt and sulfur, which may decrease Se absorption by more than 50% [94,115,121]. A number of studies have demonstrated the poor utilization/absorption of inorganic dietary Se in ruminants; absorption of inorganic ^75^Se in steers was estimated to be only 13% [122], similar true Se absorption (10–16%) was reported in non-lactating cows fed hay supplemented with inorganic Se [123,124]. Similarly, only 14% of Se consumed by cows during late pregnancy and lactation was accumulated in the body [125]. It is generally accepted that the absorption of inorganic Se in ruminants is much lower than that seen in monogastric animals [126,127], and it is likely that rumen-reducing conditions [10] and rumen micro-organisms [112] affect the metabolism and utilization of inorganic Se forms. [113,114,115]. It has been shown that significantly more Se from SS was converted to insoluble, inorganic forms by rumen microbes when compared to the use of SeMet [112]. The use of labelled Se has shown the uptake and retention of organic Se by ruminal microorganisms to be five times greater than for inorganic sources [128], which was attributed to the fact that SS is reduced to elemental Se, rendering it unavailable to the animal. Indeed, organic Se sources undergo considerably less alteration in the rumen, resulting in better availability.

However, the exact fate of SeMet in the rumen is still not well elucidated. Hidiroglou et al. [129], dosed rumen-fistulated wethers intraruminally with a single dose of ^75^Se-SeMet. Maximum ^75^Se activity in rumen fluid was found 2 h after labelled SeMet was administered. At 6 h post-dosing, 50% of the rumen liquor label was in the bacterial fraction, and only 20% of the radioactivity was recovered in the rumen at 96 h. The authors showed that majority of bacterial ^75^Se activity was in a protein-bound form. In a subsequent study, the same authors showed that 2 h post-dosing with ^75^Se-SeMet, Se-cystine, SeMet and elemental selenium could be identified in ruminal bacteria, however, 40–50% of ^75^Se activity was associated with unidentifiable components. Furthermore, the authors also showed that ^75^SeMet was metabolized by rumen bacteria to ^75^Se-selenocystine, with both selenoamino acids being incorporated into bacterial protein [129]. The study by Galbraith et al. [10] reported that the incorporation of Se into microbial mass was 13.2-fold greater for SeMet-supplemented animals when compared to un-supplemented controls, and approximately four times greater when compared with inorganic Se forms (SS and selenate), with no difference reported between selenate and SS. In addition, the formation of non-bioavailable elemental Se was shown to be lower for rumen micro-organisms incubated with SeMet when compared with inorganic Se sources. These data explain the apparent increased oral bioavailability of SeMet in ruminant animals when compared to inorganic Se sources.

However, Se availability from rumen bacterial protein needs further investigation. Bacterial Se collected from the rumen of wether sheep fed a diet supplemented with SS had lower bioavailability than in the intestine of mice when compared to mice receiving SS in mineral form [130]. In a similar study using bacterial and protozoal Se obtained from the rumen of SS-supplemented sheep, there was no difference between SS or bacterial/protozoa Se in terms of its absorption, retention and utilization in rats. However, whole blood and liver Se levels in rats fed with bacterial Se were lower than that in SS-supplemented rats, and not different from control rats fed diets without Se supplementation. None of the tested Se sources affected Se concentration in rat muscle. This suggests that the uptake and incorporation of SS into microbial protein results in the formation of Se based compounds that are subsequently unavailable to the host animal. It is not clear at present if the same issue of Se availability would be found if SeMet was used to produce Se-enriched bacteria and protozoa. No information has been found yet to show if selenite or SeMet can pass the rumen in unaltered form and be available for absorption in the small intestine and this question awaits further investigation.

Organic Se has been reported to alter rumen fermentation characteristics, feed digestion, milk yields, and milk Se [131], and the authors suggested that organic Se stimulated digestive microorganisms in a dose-dependent manner. More recently, a comparison between SS and OH-SeMet demonstrated that OH-SeMet altered rumen fermentation characteristics, improved apparent nutrient digestibility with respect to crude protein, neutral detergent and acid detergent fibre, and improved selenium absorption [132].

## 8. Beneficial Effects of Organic Selenium in Cows

The nutritional requirements of Se in cattle are estimated to be 0.1 mg/kg DM for beef cattle and 0.3 mg/kg DM for dairy cows [133], and these can be comfortably met through supplementation. However, recent reviews of the role of Se in ruminants [134,135,136] concluded that inorganic Se sources were not effective at meeting the Se requirements of ruminant animals. In general, the bioavailability of Se-Yeast has been shown to be superior to that of SS. Relative bioavailability of organic Se in the form of Se-Yeast has been shown to be 1.4 times that of SS if using blood GSH-Px as the testing criterion, 1.9 if using blood Se, and 2.7 if milk Se was used [137].

Beneficial effects of organic Se in dairy cow nutrition can be associated with improvements to health and production of both the animal and her progeny, a consequence of improved Se status. The improved Se status of the transition cow is likely to be an important factor in how the animal adapts to different stresses. At parturition, tissue nutrient reserves in the dam are likely to be at their lowest point, a consequence of the need to meet the increasing nutrient requirements of the developing fetus, as well as the need to partition nutrients into the production of colostrum and milk, which are essential to the survival and health maintenance of the newborn. However, during this time of increased nutrient partitioning, Se reserves in the dam could be compromised, resulting in a number of Se deficiency problems that are likely to affect calf viability. The replacement of SS with organic Se sources in the immediate peri-partum period have been shown to have a range of beneficial effects (Table 4), from improving the Se status of both the dam and newborn, to increasing the Se content of colostrum and milk, thus maintaining the antioxidant defenses of both the cow and her calf.

In general, organic Se can modulate antioxidant system of the dairy cow by improving enzymatic and non-enzymatic antioxidant defenses and decreasing indexes of oxidative stress. For example, cows fed Se-Yeast during the last 4 weeks of gestation were characterized by improved antioxidant status, manifested by decreased lipid peroxidation (plasma MDA concentrations) at 7 days prepartum, and at 7 and 21 days postpartum. Furthermore, there were decreased plasma ROS and H_2_O_2_ concentrations at 7 and 21 days postpartum, increased plasma and erythrocyte GSH-Px activities, and increased erythrocyte GSH concentrations at 7 days postpartum when compared to Se-adequate control cows [12]. In a separate study, OH-SeMet-supplemented cows had improved serum GSH-Px activity, total antioxidant capacity, and SOD activity when compared to SS-supplemented cows [144], and cows supplemented with OH-SeMet during a period of heat stress had increased Se concentrations in serum and milk, increased total antioxidant capacity and decreased serum MDA, hydrogen peroxide, and nitric oxide concentrations when compared with SS-fed controls [11]. The aforementioned studies clearly indicate that organic Se in cow diets upregulates antioxidant defenses. This includes increased activity of GSH-Px, an important selenoprotein playing a central role in antioxidant defence [151]. Secondly, increased concentrations of GSH could be of great importance in the regulation of redox status of tissues and the whole body [152], responsible for maintaining effective cell signaling and stress adaptation [153]. Thirdly, there was a positive effect of Se on vitamin E status showing important interactions between these two elements of the antioxidant defense systems. Fourthly was the upregulation of SOD, the central antioxidant enzyme belonging to the vitagene family [154], which could be the most important element in stress adaptation. Finally, increased antioxidant defenses led to decreased markers of oxidative stress, namely MDA, ROS and H_2_O_2_ levels. These results could be a background for understanding the role of organic Se in health maintenance of dairy cow in commercially relevant stress conditions.

There is a great body of evidence indicating that replacement of SS by organic Se in cow diets leads to a significant improvement in the Se content of milk and colostrum. Not only is this of benefit to the newborn, but it could be beneficial in meeting the Se requirements of the human population [155]. Based on a meta-analysis of 42 studies investigating the effects of oral Se supplementation on milk Se concentration in cattle, it was concluded that cows supplemented with organic Se (e.g., 6 mg/head per day for 75 days) had greater milk Se concentrations in comparison to those supplemented with inorganic forms of Se [156]. When comparing SS with Se-Yeast at 0.3 ppm, it was shown that the SeMet concentration in milk of Se-Yeast-supplemented animals increased from 61 to 111 ng Se/g, accounting for 44% of total Se, whilst the proportion of total Se comprised as SeMet in SS supplemented animals declined and was markedly lower (36 vs. 111 ng Se/g) [145].

In cows fed Se-Yeast, the efficiency of Se transfer from feed to milk has been reported to range from 9.9 to 12.5%, compared with 2.4–4.1% for those supplemented with SS [157]. A meta-analysis of milk Se data from 11 studies indicated that responses to Se-Yeast were four-fold greater than those of SS. A similar finding was found following the analysis of data from 130 individual cows looking at the efficiency of Se transfer from feed to milk [158]. Furthermore, in Se-Yeast fed cows, the amounts of Se secreted daily into milk and apparently retained in tissues was shown to increase linearly with average daily intake of Se, total Se excretion was shown to be 66%, Se secretion in milk accounted for 17%, and Se retained in tissues accounted for 17% of total Se intake [159].

An increasing body of evidence also shows that the efficiency of Se transfer to the new-born depends on the form of Se in the maternal diet. The enhanced Se status of calves born to dams that have received organic Se supplements during the latter stages of pregnancy has been reported previously [139,146,160] and is most likely attributable to SeMet transfer from the dam to the calf. Selenoprotein-based transfer mechanisms have been identified in the mouse placenta, and these ensure an adequate Se supply to the developing fetus, especially when maternal Se supply is poor [161]. However, the transport of SeMet across the placenta is unregulated and has been reported to be dependent upon maternal SeMet supply [161]. Under conditions of limited dietary Se supply, SeMet transfer could be limited and Se supply to the fetus is likely to occur through previously mentioned selenoprotein transfer mechanisms [161]. This is reflected in a study with sows, in which piglets born to SS supplemented sows had a similar Se status to that of un-supplemented controls, whereas piglets born to sows offered diets containing Se-Yeast had a much improved Se status [162]. This suggests that similar Se transfer mechanisms exists in the porcine placenta and that the enhanced Se status of piglets born to Se-Yeast supplemented sows may have been a consequence of placental SeMet transfer.

Following birth, the provision of Se to the newborn is via the ingestion of colostrum and then milk, the Se concentration of which would have been affected by the form and dose of Se the dam received in the weeks prior to parturition. A number of studies have reported the Se concentration of colostrum to be greater than that of following milk [148,163,164,165]. The reason for the difference between colostrum and milk total Se concentration remains unclear and may simply reflect differences in milk secretory processes pre- and post-partum. However, the higher Se concentration of colostrum may confer benefits to the new-born in terms of the conferment of passive immunity. For example, it was reported that passive intestinal transfer of ovalbumin was improved in calves born to Se supplemented dams [166] and IgG transfer has been shown to be better from colostrum with a higher Se content [167].

Studies investigating the effects of Se form offered in the weeks preceding birth have shown the Se content of colostrum to be higher from those animals supplemented with organic Se forms when compared to SS [164]. A number of studies have shown that the higher Se content of milk from cows fed organic Se supplements is a consequence of the incorporation of SeMet. However, very little data exists on the selenized amino acid content of colostrum. Hill et al. [168] reported that Selenoprotein P (SepP) was the major selenium transport protein in mouse milk and postulated that this may be an important mechanism in transferring maternal Se to the nursing neonate. A recent study in heifers offered OH-SeMet the elevated Se content of colostrum was a consequence of elevated SeCys rather than SeMet [9], confirming the findings of Hill et al. [168]. Given the difference in the selenized amino acid content of colostrum and milk, it could be hypothesized that the mechanisms that are responsible for IgG transfer in the new-born might also be involved in the uptake and transfer of Se as SepP in the immediate neonatal period. The advantages of this mechanism are that SepP could be transferred to the systemic circulation in very early life, thus conferring benefits to the new-born with respect to Se status. However, given the lack of data in this area, further work is needed to try and elucidate the fate of ingested Se from colostrum.

The importance of proper Se supplementation of the transition cow prior to calving cannot be overemphasized, and ingestion of colostrum by calves is critical to providing sufficient Se to neonatal calves to maintain their antioxidant defenses. Therefore, increased placental transfer of Se and its enhanced levels in colostrum could help the calf to build better Se body reserves and improve antioxidant defense to deal with aforementioned stresses. This could also potentially help improve immunity and better withstand pathogen challenges [60,98,169].

It seems likely that differences between SS and organic Se sources in dairy cattle nutrition could also be seen at the level of gene expression and transcriptomic. For example, dietary supplementation of Se in inorganic and organic forms was shown to differentially alter blood and liver Se concentrations and liver gene expression profiles of growing beef heifers [140]. In particular, both forms of supplementation appeared to upregulate mitochondrial gene expression capacity, while only organic Se supplemented animals had reduced levels of mRNA encoding oxidative-stress-related proteins. Interestingly, the form of supplemental dietary Se consumed by cattle was shown to affect the composition of liver transcriptomes, leading to different physiological capacities [170]. Importantly, the sensitivity of bovine pituitary gene expression/transcriptome (hence whole-body physiological capacities) to forms of supplemental Se has been reported [171]. Furthermore, effects of Se in the dam diet on the transcriptome of the neonatal calf testis depended on the dietary form of Se [141]. It is important to mention that Se status in ruminant mammary tissue is a vital regulator of selenoprotein activity and expression [94]. For example, SepP functions as a Se supply protein, participating in the distribution of Se from the liver to peripheral tissues being an important element of the antioxidant defence network [6]. Taking into account recent data indicating that OH-SeMet could differently modulate the selenogenome in chickens, including the expression of SepP [172], it would be of great interest to study effects of various forms of Se on the expression of this selenoprotein in ruminants and to determine the effect of supplemental dietary Se forms on the expression of the SelP receptors (megalin/LRP2, APOER2/LRP8) and the phenomenon of hierarchical tissue assimilation of Se. Furthermore, evidence is quickly accumulated to prove that organic Se in maternal diet could have a long-term benefit on the intake of steer progeny, and this could lead to improvements in animal growth [173]. There is growing body of evidence indicating that maternal Se can affect progeny performance. For example, calf birthweight was significantly increased with maternal supplementation of Se-Yeast (0.3 ppm), but growth to day 56 was not affected by the methods of Se supplementation [174]. Similarly, average daily gain in calves born from Se-Yeast supplemented cows (0.5 ppm) tended to be higher than in SS at 0.5 ppm and SS at 0.1 ppm [146]. Further studies in this area are needed to elucidate molecular mechanisms of the maternal effect of Se in dairy cows.

It should be stated that in most of the studies mentioned above, replacing SS with an organic source of Se in diets adequate in basal Se concentrations did not affect Se status, uterine health, fertilization, or embryo quality in early lactation of dairy cows [175]. Indeed, cow performance, milk production, or reproductive efficiency did not depend on dietary Se source. Organic Se was shown to decrease milk protein and increase milk lactose but did not affect the pre-weaning performance of progeny from Se-adequate cows [176]. The aforementioned data indicate that organic Se in dairy cattle nutrition is an investment in an insurance policy to ensure optimal cow/calf performance [94,177]. Indeed, when stress is low, Se requirement, in many cases, can be met by feed-derived Se and additional Se supplementation would be unlikely to improve animal productive and/or reproductive performance. However, under stress conditions, when Se requirements would increase but feed consumption may decline, additional organic Se supplementation would help maintain antioxidant defenses and animal health [6]. Indeed, building Se reserves in muscles as a result of organic Se supplementation would improve animal adaptive ability to stress by improving selenoprotein synthesis in commercially relevant stress-conditions [6,103].

## 9. Conclusions

Organic Se has been shown to be more available to dairy cows when compared to inorganic forms. This difference is primarily a consequence of rumen reducing conditions where microbes reduce inorganic Se forms to metallic non-available forms. In such conditions, organic Se is less affected and becomes incorporated into microbial protein. However, the ultimate fate of Se in rumen and post-rumen is still unknown and requires further research. It has been shown that oxidative stress during the periparturient and early lactation period contributes to a number of health disorders in dairy cattle. Among feed-derived antioxidants, Se is essential as it is a component of the 25 selenoproteins identified in animals. Organic Se has been shown to significantly increase the Se concentration in body tissues of ruminant animals [99,178], which could be used as a reserve in times of Se deficit or elevated stress. Consequently, the use of organic Se in dairy cattle nutrition could be beneficial, especially during transition stress [179] and heat stress [11]. In general, the beneficial effects of using organic Se were demonstrated in a wide variety of species, including chickens [180], turkeys [181], pheasants [182], pigs [8], beef cattle [140,142,149,183,184], sheep [185,186,187], goats [188,189], and horses [190,191].

From the data presented, it is clear that organic Se, in the form of SeMet, is a natural form of Se, and during evolution the digestive system of animals adapted to utilize this form of Se [103]. It seems likely that building Se reserves in the body is an important adaptive mechanism to deal with stressful conditions, and since livestock production is associated with a range of unavoidable stresses [6,49], optimal nutrition using balanced diets supplemented with optimal doses of micronutrients in their most effective forms is considered to be an essential part of precision nutrition. Indeed, organic Se is an important part of this concept and it is likely that over time the use of SS in feed premixes will be replaced by organic forms of Se. However, there is a need for more studies with dairy herds housed in realistic commercial conditions that reflect potentially stressful situations to demonstrate the beneficial/protective effects of organic Se, and to justify any increased cost of supplying organic forms of supplemental Se.

## Figures and Tables

**Table 1:** Biologically important reactive oxygen and nitrogen species (Adapted from Halliwell and Gutteridge [14]).

Free Radicals	Non-Radicals
**Reactive Oxygen Species (ROS)**	
Superoxide, O_2_*^−^	Hydrogen peroxide, H_2_O_2_
Hydroxyl, OH*	Organic peroxides, ROOH
Hydroperoxyl, HO_2_*	Peroxinitrite, ONOO^−^
Peroxyl, RO_2_*	Hypochlorous acid, HOCl
Alkoxyl, RO*	Hypobromous acid, HOBr
**Reactive Nitrogen Species (RNS)**	
Nitric oxide, NO*	Nitrous acid, HNO_2_
Nitrogen dioxide, NO_2_*	Dinitrogen trioxide, N_2_O_3_
Nitrate radical, NO_3_*	Dinitrogen tetroxide, N_2_O_4_

**Table 2:** Advantages and disadvantages of various biomarkers as indicators of oxidative stress in ruminants (Adapted from [50,51,52,53,54,55,56,57,58]).

Biomarker	Advantages	Disadvantages
MDA	Sensitive and reproducible	Non-specific product of lipid peroxidation
TBARS	Rapid, popular, easy, and economical	Non-specific, non-reproducible, no quantitative relationship with lipid peroxidation
F2-Isoprostane	Specific, reproducible, sensitive	Expensive, auto-oxidation of samples, sample derivatisation is required
ORAC	Sensitive and covers a wide variety of antioxidants	Requires spectrofluorometer; AAPH, a free radical source is sensitive to temperature, low reactivity of fluorescein toward ROO• radicals
FRAP	inexpensive, reagents are simple to prepare, results are highly reproducible, and the procedure is straightforward and speedy	The reaction is non-specific, and the result of the test depends on the reaction time.
TEAC	Extremely fast and simple	Results vary with sample dilution; antioxidant used may interact with solvent molecules; specificity varies
TRAP	Gives an idea of the rate of free radical formation	Antioxidant employed may not trap all types of free radicals
ROMs	Extremely fast, simple; can be performed directly in whole blood, inflammatory fluids, cell extracts and respiratory condensate	Inhibited by sodium azide, lack of reference values
RONS	Fast, commercial Kits are available	lack of reference values
BAP	fast, simple and covers a wide variety of antioxidants	Can be performed only in plasma and serum samples; hyperlipemic samples can underestimate results
AOPPs	Novel markers of protein oxidation, quickly developing, mediators of pro-inflammatory response	lack of reference values
Protein carbonylation	Easy to perform	lack of reference values
AO enzymes (SOD, GSH-Px, Catalase, etc.)	Common, widely used tests, commercial kits are available	Difficulties with results interpretation, since some enzymes are stress-inducible
Plasma total thiols	Important part of the Redox system, commercial kits are available	Very sensitive to oxidation during sample preparation and storage
Non-enzymatic antioxidants: glutathione, α-tocopherol, β-carotene, uric acid, etc.	Common, widely used tests.	Individually reflect only a small proportion of the antioxidant defence potential
HSP	Important elements of antistress protection	Difficult to perform, difficulties with results interpretation, since HSP are stress-inducible

AAPH, 2,2′-azobis (2-amidinopropane) dihydrochloride; AGE, advanced glycation end products; AOPPs, Advanced oxidation protein products; BAP, biological antioxidant potential; FRAP, ferric reducing ability of plasma; HSP, heat shock proteins; MDA, malondialdehyde; ORAC, oxygen radical absorbance capacity; ROMs, reactive oxygen metabolites; RONS, reactive oxygen and nitrogen species; TBARS, thiobarbituric acid reactive substances; TEAC, Trolox equivalent antioxidant capacity; TRAP, total radical antioxidant potential.

**Table 3:** Important effectors of oxidative stress in dairy cows.

Conditions	Markers	References
Biological/Metabolic Stresses		
Periparturient dairy cow	Plasma ROS + RNS↑, AOA↓, OSi↑, 15-F2t-isoprostane↑, TBARS↑, hydroperoxides↑	[62,63]
Dairy cow at the end of the first week (Day 7) after parturition	GSH↓, GSH-Px↓, CAT↓, vitamin E↓, T-AOC↓, ROS↑, H2O2↑, MDA↑	[12,64]
Nutritional Stresses		
Dairy cows with body weight and body condition increase due to a ration of increasing energy density for 15 wk	dROM↑, TBARS↑	[53]
Dairy cows in severe negative energy balance during early lactation	BAP↓	[65]
Fish oil-fed dairy cows	Plasma MDA↑, AST↑, ALP↑	[66]
Dairy cows fed AFB1-contaminated diets	MDA↑, SOD↓, GSH-Px↓, T-AOA↓	[67]
Environmental Stresses		
Heat stress in late-pregnant dairy cows	MDA↑, cortisol↑, Nrf2-mediated oxidative stress response↑	[68]
Heat stress in postpartum Holstein cows	Oxidative phosphorylation↑, mitochondria disfunction↑, Nrf2-mediated oxidative stress response↑	[69]
Pathogen/Disease Stresses		
Dairy cows naturally infected with the lungworm Dictyocaulus viviparus (Nematoda: Trichostrongyloidea).	TBARS↑, ROS↑, SOD↑, CAT↓	[70]
Dairy cows seropositive and symptomatic for Neospora caninum	serum ROS↑, TBARS↑, NO↑, GST↓, T-AOA↓	[71,72]
Ketotic dairy cows	plasma SOD↓, CAT↓, vitamin C↓, vitamin E↓, hydroxyl radical capacity↓, H2O2↑, MDA↑	[73]
Dairy cows with Grade 2 Endometritis	AOOP↑	[74]

**Table 4:** Advances of organic selenium for ruminants.

Parameter	Effect of Organic vs. Inorganic Selenium	References
Se in cow plasma	Increased	[138]
Se in cow serum	Increased	[11]
Se in cow whole blood	Increased	[139,140,141,142,143]
Se in cow milk	Increased	[138,143,144,145,146,147]
Se in cow whole blood, red blood cells and liver	Increased	[140]
Se in cow cheese	Increased	[145]
SeMet in cow milk	Increased	[145]
Se in cow colostrum	Increased	[9,148]
Se in heart, kidney and muscle of beef cattle	Increased	[142]
Se in whole blood of calves at birth	Increased	[149]
Se in whole blood of calves	Increased	[149]
Se in plasma of calves	Increased	[148]
GSH-Px in serum of cows	Increased	[143]
GSH-Px in whole blood of cows	Increased	[142,148]
GSH-Px in erythrocytes of calves at birth	Increased	[149]
SelP in serum of cows	Increased	[143]
TrxR in serum of cows	Increased	[143]
Total AOA in serum of cows	Increased	[143]
Catalase in serum of cows	Increased	[143]
IL1 in serum of cows	Increased	[143]
IgA in serum of cows	Increased	[143]
Somatic cell counts	Decreased	[150]
Fat in milk	Increased	[150]

## Abbreviations

AP1	activator protein 1 (a transcription factor)
BD	basic diet
DM	dry matter
GSH	reduced glutathione
GSH-Px	glutathione peroxidase
4-HNE	4-hydroxyalkenal
MAPK	mitogen-activated protein kinase
MDA	malondialdehyde
Met	methionine
NF-κB	nuclear factor-kappa B (a transcription factor)
Nrf2	NF-E2-related factor 2 (a transcription factor)
OS	oxidative stress
OSi	oxidative stress index
PMN	polymorphonuclear neutrophil
PPAR	peroxisome proliferator-activated receptor
PUFA	polyunsaturated fatty acids
PRRs	pathogen recognition receptors
RNS	reactive nitrogen species
ROS	reactive oxygen species
RP	retained placenta
SeCys	selenocysteine
SeMet	selenomethionine
SepP	selenoprotein P
Se-Yeast	selenized yeast
SOD	superoxide dismutase
SS	sodium selenite
TLR	Toll-like receptor
